# Design and characterization of an urea-bridged PMO supporting Cu(II) nanoparticles as highly efficient heterogeneous catalyst for synthesis of tetrazole derivatives

**DOI:** 10.1038/s41598-022-22905-7

**Published:** 2022-10-28

**Authors:** Ehsan Valiey, Mohammad G. Dekamin

**Affiliations:** Pharmaceutical and Heterocyclic Compounds Research Laboratory, Department of Chemistry, Iran University of Science and Technology, Tehran, 16846-13114 Iran

**Keywords:** Environmental chemistry, Catalysis, Coordination chemistry, Green chemistry, Inorganic chemistry, Materials chemistry, Organic chemistry, Polymer chemistry, Supramolecular chemistry, Chemical synthesis

## Abstract

In this work, a new periodic mesoporous organosilica with urea-bridges produced by the reaction of (3-aminopropyl)triethoxysilane and toluene-2,4-diisocyanate (APS-TDU-PMO) is introduced. The obtained APS-TDU-PMO was found to be an appropriate support for loading of Cu(II) nanoparticles to afford supramolecular Cu@APS-TDU-PMO nanocomposite. Uniformity and mesoporosity of both synthesized nanomaterials including APS-TDU-PMO and Cu@APS-TDU-PMO were proved by different spectroscopic, microscopic or analytical techniques including FTIR, EDX, XRD, FESEM, TEM, BET, TGA and DTA. Furthermore, the prepared Cu@APS-TDU-PMO nanomaterial was also used, as a heterogeneous and recyclable catalyst, for the synthesis of tetrazole derivatives through cascade condensation, concerted cycloaddition and tautomerization reactions. Indeed, the main advantages of this Cu@APS-TDU-PMO is its simple preparation and high catalytic activity as well as proper surface area which enable it to work under solvent-free conditions. Also, the introduced Cu@APS-TDU-PMO heterogeneous catalyst showed good stability and reusability for six consecutive runs to address more green chemistry principles.

## Introduction

Mesoporous silica nanomaterials (MSNs) are widely used in material science for various applications as well as special blocks for making various valuable assemblies. MSNs have been extensively studied and used for applications in diverse fields including catalysis, ion exchange, adsorption, chromatography, molecular sieving, CO_2_ capture and even as templates for synthetic conductive carbon nanowires^1–22^ due to their special properties such as large specific surface, high pore volume, uniform and tunable pores, high stability, and low cost^23–30^. Among different MSNs, periodic mesoporous organosilicas (PMOs) have received significant research interest in recent years. PMOs are hybrid porous materials with high surface area and large porosity obtained by sol–gel method from an organo-bridged alkoxysilane in the presence of a surfactant, which were first reported in 1999^31–44^. The porous framework of PMOs is created by organic functional groups covalently binding to siloxane domains. In contrary to SBA-15 and MCM-41 which can only be functionalized at their surfaces by the grafting method^45,46^, different organic functionalities of the organic-bridged silica precursors can be included in the silica framework^47–49^. PMOs have some advantages over mesoporous silica materials including mechanical stability, hydrophobic pore wall, and high concentration of organic functional group in the framework, which have led to their applications in different fields such as hydrophobic drug carriers^50^, adsorbents^51,52^, biological/biomedical supports^53^, optical applications^54^, solid chromatographic phases^55^ and catalysis^44,56–60^.

Nowadays, developing of more efficient catalytic systems for the synthesis and manufacturing of both fine and bulk chemicals is in the focal point of academic as well as industrial research groups^61–63^. new commercial applications, while environmental legislation created market pull to use catalysis to meet the new regulatory standards. As we move forward into the new century, we continue to see market pull from growing interests in biomass, sustainability, emissions control, and energy. due to It is essential to use suitable catalytic systems for the preparation of very important medically and ecologically compounds^64,65^. It is essential to use suitable catalytic systems for the preparation medically and ecologically very important compounds^64,65^. Therefore, catalytic systems can be used for these purposes. The catalytic system consists of homogeneous and heterogeneous types. Also, heterogeneous catalysts have received more attention due to their many advantages such as easy separation from the reaction mixture, recyclability and subsequent reusability as well as less contamination in the final product^66,67^. In this context, nanomaterials demonstrate appropriate selectivity in activity and shape due to their finely porous structure and high surface area^68^. Also, by incorporation of acid and base centers as well as transition metallic species, they can be used in many organic transformations including acid or base-catalyzed reactions, oxidation, C–C coupling reactions, C–H activation, etc. to afford simple or complex molecules in both bulk and fine chemicals synthesis^69–73^. Among the various metal nanoparticles, copper nanoparticles are of particular importance due to their high conductivity and natural abundance, easy access, low cost, and tremendous copper potential to replace precious metals such as palladium, platinum, gold, or silver. Indeed, immobilization of copper(II) species on organic and inorganic supports bearing appropriate ligands is one of the best methods for the production of heterogeneous catalytic systems with high stability, activity, and loading of active centers^74–76^. Therefore, catalytic systems can be used for these purposes. The catalytic system consists of homogeneous and heterogeneous types. Also, heterogeneous catalysts have received more attention due to their many advantages such as easy separation from the reaction mixture, recyclability and subsequent reusability as well as less contamination in the final product^66,67^. In this context, nanomaterials demonstrate appropriate selectivity in activity and shape due to their finely porous structure and high surface area^68^. Also, by incorporation of acid and base centers as well as transition metallic species, they can be used in many organic transformations including acid or base-catalyzed reactions, oxidation, C–C coupling reactions, C–H activation, etc. to afford simple or complex molecules in both bulk and fine chemicals synthesis^69–73,77,78^. Among the various metal nanoparticles, copper nanoparticles are of particular importance due to their high conductivity and natural abundance, easy access, low cost, and tremendous copper potential to replace precious metals such as palladium, platinum, gold, or silver. Indeed, immobilization of copper(II) species on organic or inorganic supports bearing appropriate ligands is one of the best methods for the production of heterogeneous catalytic systems with high stability, activity, and loading of active centers^74–76^.

**Figure 1:**
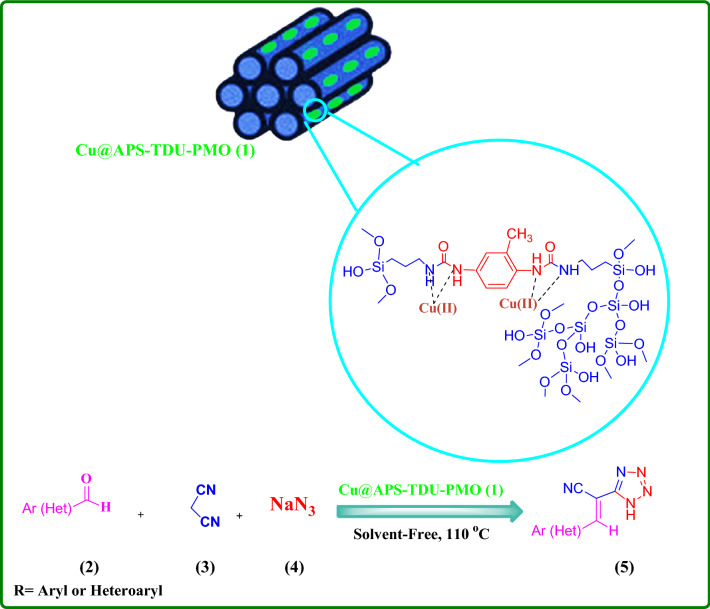
Schematic structure of the Cu@APS-TDU-PMO nanoreactor (**1**) for the three-component condensation of aldehydes (**2a–i**), malononitrile (**3**), and sodium azide (**4**).

Due to the use of copper complexes in various chemical reactions such as hydroboration of alkenes^79,80^, β-boration of α,β-unsaturated esters^81^, direct addition of terminal alkynes to imines^82,83^, alkyne-azide cycloaddition^84,85^ and allylic alkylation reactions^86^, special attention has been paid to copper complexes as catalysts for these reactions. Furthermore, the Cu(I)-assisted click chemistry (CuACC) of azide–nitrile cycloaddition for synthesis of corresponding heterocyclic compounds, namely tetrazole derivatives, has received much attention in recent years^87–91^. Tetrazole derivatives are an important class of nitrogen-rich heterocyclic nucleus with significant applications in the medicinal chemistry. Indeed, tetrazole moiety serves as a useful pharmacophore in brand names including Lasortan, Irbesartan, and Tomelukast medications, which are used to treat high blood pressure, heart failure, diabetic kidney or asthma diseases. Furthermore, this pharmacophore is widely used in drug design studies as an appropriate isostere of carboxylic acid functional group^92–99^. In addition, application of tetrazole derivatives as important widespread compounds in synthetic organic chemistry^100,101^, catalysis and energetic applications^102^, materials chemistry^103^, and as ligands in coordination chemistry^104^ have led to the development of various efficient synthetic methods. Tetrazole derivatives are most commonly synthesized by [3 + 2] cycloaddition reaction of the corresponding azide and nitrile moieties^105–112^. Therefore, attempts for the production of tetrazole derivatives through new methods have led to the use of 2-benzylidenemalononitrile and sodium azide along these lines^113,114^. In continuation of interest to develop new PMOs or their assemblies and exploring their catalytic activities^21,22,30,59,60,114^, this paper reports the synthesis of novel periodic mesoporous organosilica by the reaction of amino group of (3-aminopropyl)triethoxysilane and toluene-2,4-diisocyanate, namely APS-TDU-PMO, with walls having urea bridges for Cu nanoparticles loading (Cu@APS-TDU-PMO, **1**). Furthermore, the Cu@APS-TDU-PMO was used as a highly efficient and recoverable nanoreactor for the synthesis of tetrazole derivatives **5** via three-component addition of aldehydes (**2**), malononitrile (**3**), and sodium azide (**4**) (Fig. 1).

## Results and discussion

**Figure 2:**
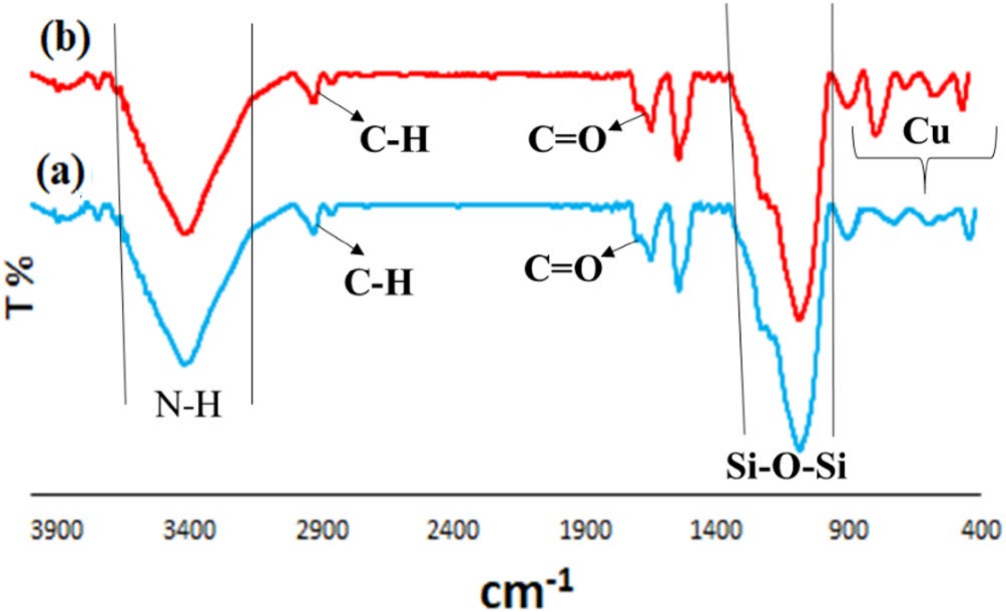
FTIR spectra of the APS-TDU-PMO (**a**) and Cu@APS-TDU-PMO (**1**, **b**).

The prepared Cu@APS-TDU-PMO nanomaterial (**1**) was characterized by Fourier transform Infrared (FTIR), thermal gravimetric analysis (TGA), differential thermal analysis (DTA), field emission scanning electron microscopy (FESEM), transmission electron microscopy (TEM), energy-dispersive X-ray (EDX), X-ray powder diffraction (XRD), and Brunauer–Emmett–Teller (BET) surface area analytical methods or techniques. The FTIR spectrum of both new APS-TDU-PMO and Cu@APS-TDU-PMO (**1**) are shown in Fig. 2. The broad absorption band at 3414 cm^−1^ is attributed to both N–H and O–H bonds stretching vibrations. Furthermore, two sharp absorption signals at 2928 cm^−1^ and 2862 cm^−1^ are assigned to the asymmetric and symmetric stretching vibration of aliphatic C − H bonds, respectively. On the other hand, the absorption bands at 1682 cm^−1^ and 1654 cm^−1^ correspond to C=O bond stretching vibration of the urea moiety in the structure of APS-TDU-PMO and Cu@APS-TDU-PMO (**1**). Furthermore, the band at 1544 cm^−1^ can be assigned to the stretching vibration of the aromatic C=C bonds. Also, two absorption bands at 1192 cm^−1^ and 1092 cm^−1^ are related to the Si–O–Si bonds (Fig. 2a). Interestingly, the absorption band of Cu–N chelation is clearly observed at the range of 800–700 cm^−1^ (Fig. 2b).

**Figure 3:**
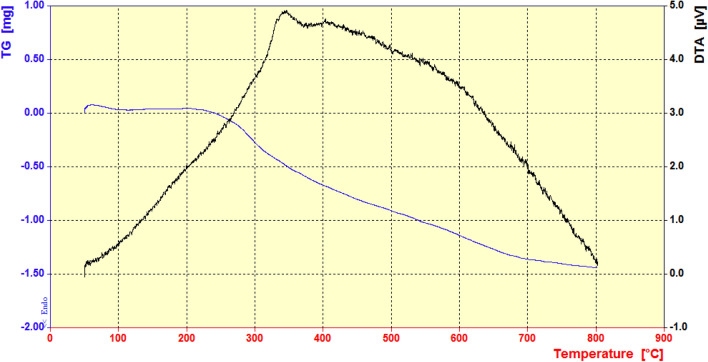
TGA and DTA curves of the Cu@APS-TDU-PMO nanomaterial (**1**).

On the other hand, TGA and DTA curves of the Cu@APS-TDU-PMO (**1**) in Fig. 3 shows that the slight weight loss between 50 and 270 °C can be assigned to the elimination of adsorbed solvent or water molecules on its surface as well as degradation of small amounts of the unextracted surfactant (P123). Also, the weight loss between 270 and 460 °C is attributed to the decomposition of the urea bridges in the Cu@APS-TDU-PMO (**1**) structure. On the other hand, the last step of weight loss between 460 and 800 °C is due to condensation of the silanols to siloxanes in the structure of Cu@APS-TDU-PMO nanomaterial (**1**).

**Figure 4:**
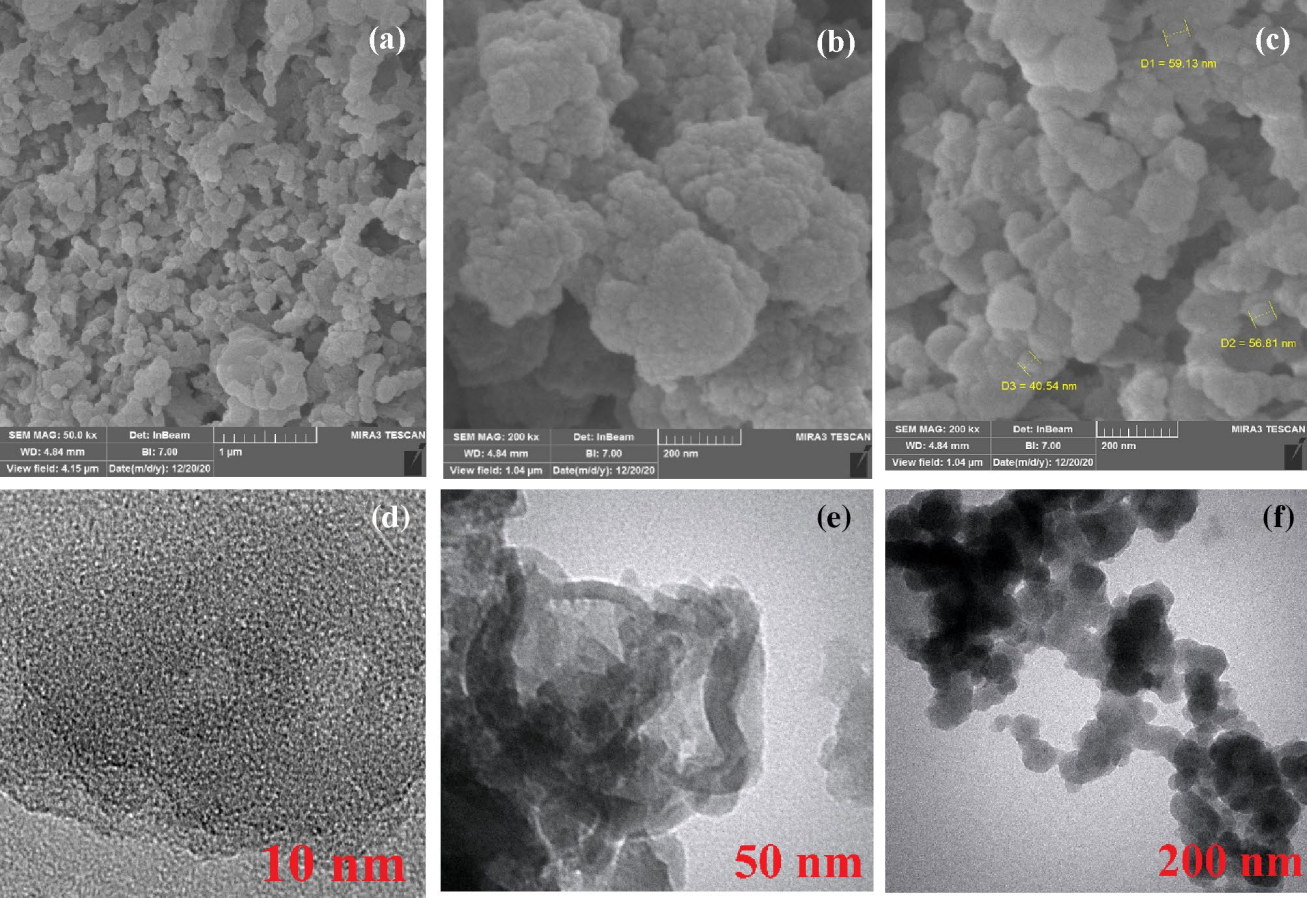
FESEM (**a**–**c**) and TEM images (**d**–**f**) of the Cu@APS-TDU-PMO nanoreactor (**1**).

Furthermore, FESEM and TEM images show that the Cu@APS-TDU-PMO nanomaterial (**1**) is composed of a large number of interwoven rods with 41–59 nm in width (Fig. 4). It can also be seen that the morphology of PMO was mostly preserved after deposition of Cu nanoparticles. TEM images also demonstrate the uniform arrangement of mesopores and tubular mesochannels in the Cu@APS-TDU-PMO (**1**) structure (Fig. 4b).

**Figure 5:**
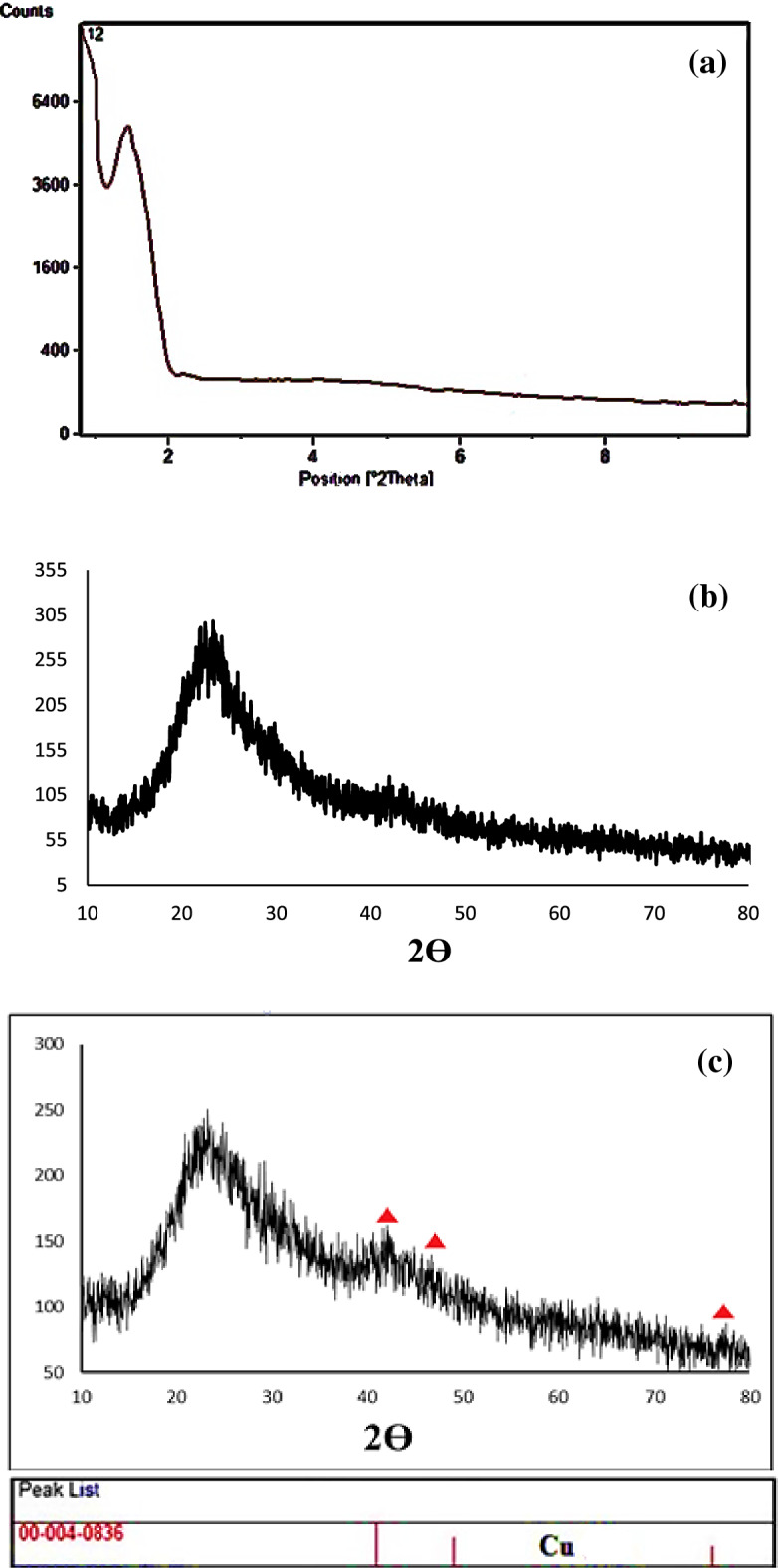
Low-angle (**a**) and wide angle (**b**) XRD patterns of the APS-TDU-PMO; Wide angle XRD pattern of the Cu@APS-TDU-PMO (**1, c**).

There is a peak at 2θ = 1.35° in the low-angle XRD pattern, indicating the mesoporous structure of Cu@APS-TDU-PMO (**1**, Fig. 5a)^115^. Also, the wide-angle diffraction signal at 2θ of 20–30°, which is characteristic of mesoporous structures, is observed in the wide-angle XRD patterns of APS-TDU-PMO and Cu@APS-TDU-PMO (**1**, Fig. 5b,c)^116^. The diffraction peaks at 2θ of 44.30°, 50.30°, and 77.50° can be assigned to the reflections of Cu(II) species nanoparticles coordinated to the surface of APS-TDU-PMO^117^ (JCPDS card No. 00–004-0836, marked with ▲).

**Figure 6:**
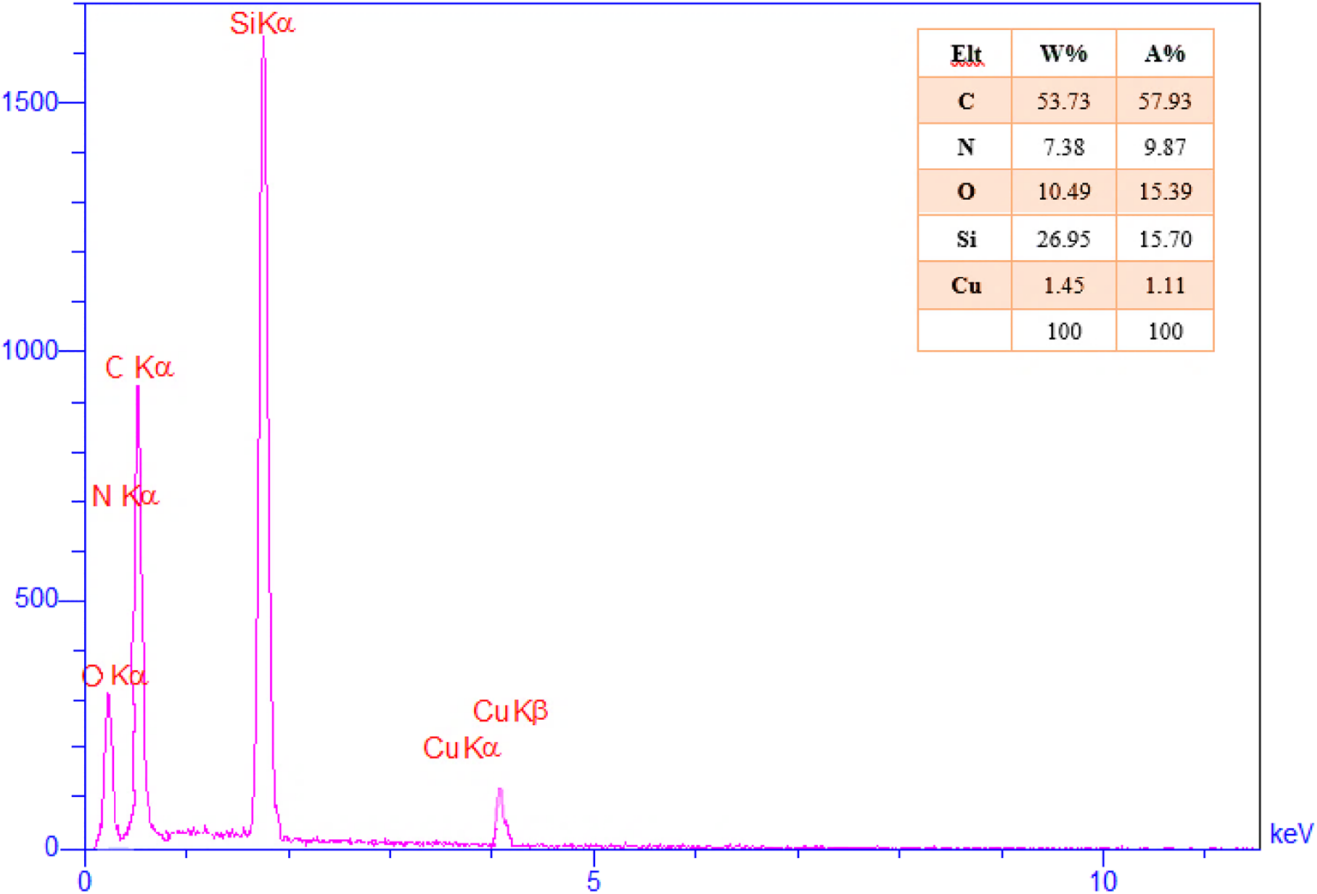
EDX analysis of the Cu@APS-TDU-PMO nanocomposite (**1**).

Also, EDX analysis confirmed the presence of C, N, O, Si, and Cu elements in the composition of Cu@APS-TDU-PMO (**1**) nanocomposite (Fig. 6).

**Figure 7:**
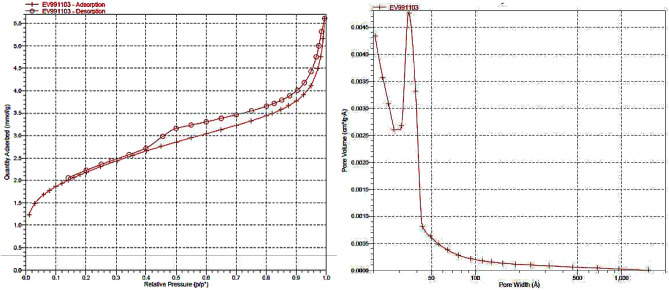
N_2_ adsorption–desorption isotherm of the Cu@APS-TDU-PMO mesoporous material (**1**).

On the other hand, the N_2_ adsorption–desorption isotherm for Cu@APS-TDU-PMO nanomaterial (1) represented type IV isotherm (Fig. 7), which is commonly observed for mesoporous silica structures. The calculated BET surface area was approximately 276 m^2^ g^−1^ which was retained even after the deposition of Cu(II) nanoparticles. The average pore size was about 5.74 nm (Table 1).

**Table 1:** Structural parameters of the Cu@APS-TDU-PMO (**1**) determined from N_2_ adsorption–desorption experiment.

Sample	Pore diameter (nm)	Surface area (m^2^ g^−1^)	Vp (cm^3^ g^−1^)
Cu@APS-TDU-PMO (**1**)	5.74	276	0.17

### Catalytic application of the Cu@APS-TDU-PMO nanomaterial (**1**) for the synthesis of 2-(1*H*-tetrazol-5-yl) acrylonitrile derivatives **5a–l**

The catalytic performance of the prepared Cu@APS-TDU-PMO nanocomposite (**1**) was investigated for the synthesis of 2-(1*H*-tetrazol-5-yl) acrylonitrile derivatives in the next step of our study. To determine the optimal reaction conditions, the three-component reaction of benzaldehyde (**2a**), malononitrile (**3**) and sodium azide (**4**) was selected as the model reaction. The model reaction was investigated in different solvents at various temperatures and catalyst loadings. The optimized reaction conditions are shown in Table 2. Initially, the model reaction was performed under different conditions without any catalyst. The obtained results showed that the reaction efficiency to afford desired (*E*)-3-phenyl-2-(1*H*-tetrazol-5-yl)acrylonitrile product **5a** was negligible or low even after 120 min (Entries 1–7). Then, the model reaction was performed in EtOH and DMF as well as under solvent-free conditions in the presence of 50 mg of both APS-TDU-PMO catalysts (Entries 8–11). The model reaction was also studied at different temperatures to find 110 °C under solvent-free conditions as the optimal temperature (Entries 11–13). The results also indicated that the optimal amount of Cu@APS-TDU-PMO nanocomposite catalyst (**1**) loading is 30 mg for the model reaction. Indeed, lower amounts of catalyst loadings led to reduced efficiency of the model reaction (Entries 14–16). In order to prove the heterogeneous nature of the Cu@APS-TDU-PMO catalyst (**1**), hot filtration experiment was performed. For this purpose, the Cu@APS-TDU-PMO catalyst (**1**) was separated from the reaction mixture after 20 min. Then, the reaction continued in the absence of nanocatalyst (**1**) for another 30 min. No further increase in the conversion of benzaldehyde (**2a**) was observed, confirming presence of Cu(II) active sites for the synthesis of 2-(1*H*-tetrazol-5-yl)acrylonitrile derivative **5a** on the surface of Cu@APS-TDU-PMO nanocatalyst (**1**). Also, no effect of copper release was observed in the reaction mixture, which is well indicated by FTIR and XRD pattern analysis of the recycled catalyst **1**.

**Table 2:** Screening of optimal conditions for the synthesis of (*E*)-3-phenyl-2-(1*H*-tetrazol-5-yl)acrylonitrile product **5a**.


Entry	Catalyst	Catalyst loading (mg, mmol Cu(II))	Solvent	Temperature (°C)	Time (min)	Yield 5a^a^ (%)	TON	TOF (h^−1^)
1	–	–	Solvent-free	r.t.	120	Trace	0	0
2	–	–	H_2_O	r.t.	120	Trace	0	0
3	–	–	DMF	r.t.	120	30	0	0
4	–	–	EtOH	r.t.	120	20	0	0
5	–	–	EtOH	Reflux	120	20	0	0
6	–	–	DMF	Reflux	120	40	0	0
7	–	–	Solvent-free	110	120	45	0	0
8	APS-TDU-PMO	50	Solvent-free	110	50	30	–	–
9	Cu@APS-TDU-PMO (**1**)	50 (1.141 × 10^–3^)	EtOH	Reflux	50	38	333	400
10	Cu@APS-TDU-PMO (**1**)	50 (1.141 × 10^–3^)	DMF	Reflux	50	65	570	684
11	Cu@APS-TDU-PMO (**1**)	50 (1.141 × 10^–3^)	Solvent-free	110	50	90	789	947
12	Cu@APS-TDU-PMO (**1**)	50 (1.141 × 10^–3^)	Solvent-free	r.t.	50	Trace	–	–
13	Cu@APS-TDU-PMO (**1**)	50 (1.141 × 10^–3^)	Solvent-free	80	50	40	351	421
14	Cu@APS-TDU-PMO (**1**)	30 (0.685 × 10^–3^)	Solvent-Free	110	50	97	1416	1699
15	Cu@APS-TDU-PMO (**1**)	20 (0.459 × 10^–3^)	Solvent-free	110	50	70	1525	1830
16	Cu@APS-TDU-PMO (**1**)	10 (0.228 × 10^–3^)	Solvent-free	110	50	55	2412	2895

Reaction conditions: benzaldehyde (**2a**, 1.0 mmol), malononitrile (**3**, 1.0 mmol), sodium azide (**4**, 1.2 mmol) and Cu@APS-TDU-PMO (**1**) under different conditions and solvent (2 mL, if not otherwise stated).

^a^Isolated yield.

In the next step, 30 mg of the Cu@APS-TDU-PMO nanoreactor (**1**) under solvent-free conditions at 110 °C was selected as the optimal reaction conditions for the synthesis of other 2-(1*H*-tetrazol-5-yl) acrylonitrile derivatives. Various aromatic aldehydes with electron-withdrawing or electron-donating groups (entries 1–10) as well as heteroaromatic aldehydes (entries 11, 12) were involved in the optimal reaction conditions to afford the corresponding (*E*)-3-aryl/heteroyl-2-(1*H*-tetrazol-5-yl) acrylonitrile derivatives **5b–l** in high to quantitative yields. In fact, aldehydes having electron-withdrawing substitutions react more rapidly and have higher efficiencies than aldehydes containing electron-releasing groups. These observations indicate that formation of the Knoevenagel condensation intermediate may be rate-determining step of this three-component reaction. The obtained results are summarized in Table 3.

**Table 3:** Scope of the synthesis of different 2-(1*H*-tetrazol-5-yl) acrylonitrile derivatives **5a–l** catalysed by Cu@APS-TDU-PMO nanoreactor (**1**).

Entry	Substrate (2)	Product	Time (min)	Yield (%)	Mp (°C)	
					Observed	Reference
1			50	97	168–169	170–171^118^
2			52	93	174–176	175–177^119^
3			55	92	189–191	189–191^118^
4			60	89	158–160	159–161^118^
5			60	91	167–168	166–168^120^
6			56	93	177–179	176–179^119^
7			53	92	152–153	153–155^118^
8			56	89	164–166	165–167^120^
9			52	92	143–145	142–143^120^
10			40	95	166–168	165–168^118^
11			56	90	87–88	85–86^120^
12			55	93	252–254	253–254^120^

Reaction conditions: aldehydes (**2**, 1 mmol), malononitrile (**3**, 1 mmol), sodium azide (**4**, 1.2 mmol) and Cu@APS-TDU-PMO (**1**, 0.03 g) under solvent-free conditions conditions.

### The proposed mechanism for the synthesis of 2-(1*H*-tetrazol-5-yl) acrylonitrile derivatives 5 catalysed by Cu@APS-TDU-PMO nanocatalyst (**1**)

**Figure 8:**
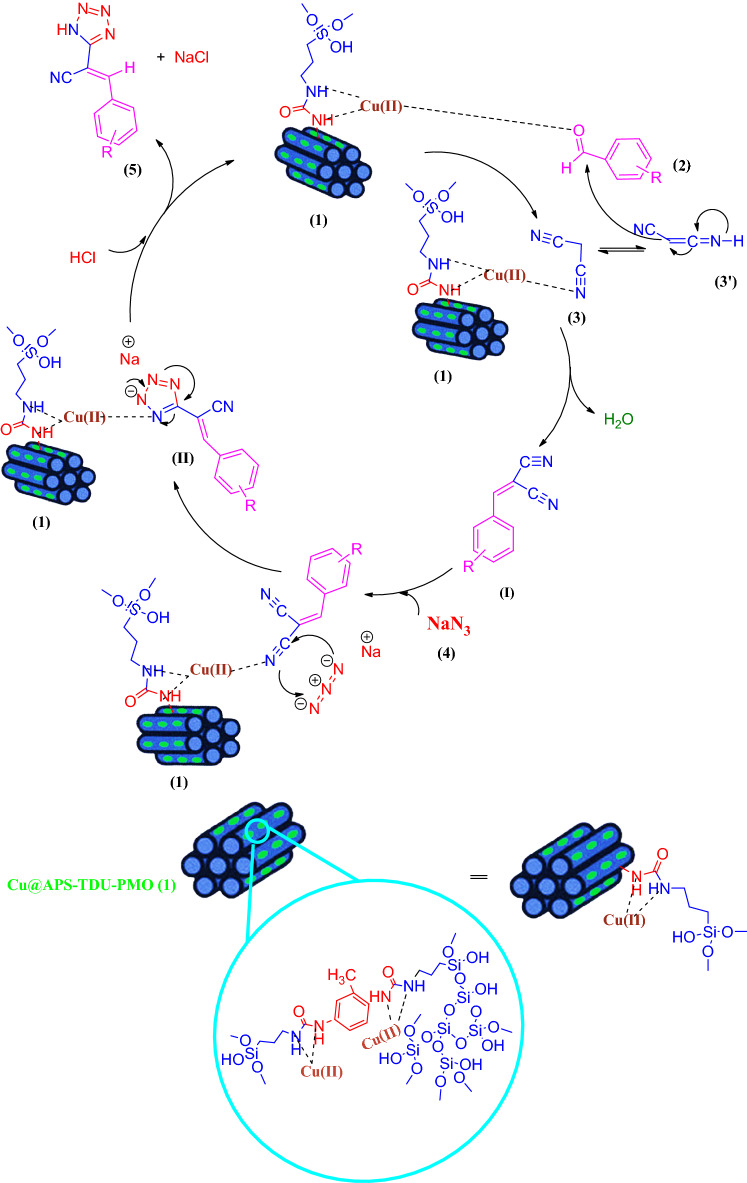
The proposed mechanism for the synthesis of 2-(1*H*-tetrazol-5-yl) acrylonitrile derivatives in the presence of Cu@APS-TDU-PMO nanoreactor (**1**).

A plausible mechanism for the synthesis of 2-(1*H*-tetrazol-5-yl) acrylonitrile derivatives in the presence of Cu@APS-TDU-PMO nanocatalyst (**1**) is shown in Fig. 8. Initially, the carbonyl group of aromatic aldehyde **2** and the nitrile group of malononitrile (**3**) are activated by the Cu@APS-TDU-PMO catalyst (**1**) to afford the Knoevenagel condensation intermediate (**I**). Then, Cu(II) species of the catalyst **1** activates one of the C≡N functional groups of the intermediate (**I**) to promote [3 + 2] cycloaddition reaction between it and sodium azide (**4**) and producing intermediate (**II**). In the next step, sodium salt of 2-(1*H*-tetrazol-5-yl) acrylonitrile derivative, as a more desirable tautomer in condensed media^121^, can be formed via the tautomerization of intermediate (**II**). Subsequently, the catalyst **1** is separated from the sodium salt of more stable tautomer using an aqueous solution of HCl to afford product **5**.

### Comparison of the catalytic activity of Cu@APS-TDU-PMO (1) catalyst for the synthesis of 2-(1*H*-tetrazol-5-yl) acrylonitrile derivative **5**

To show merits of this mew methodology, Table 4 compares the catalytic activity of Cu@APS-TDU-PMO (**1**) with some similar catalytic systems reported in the literature for the synthesis of (*E*)-3-phenyl-2-(1*H*-tetrazol-5-yl)acrylonitrile derivative **5a** in terms of the active catalytic sites or used support. In fact, specific advantages of Cu@APS-TDU-PMO catalyst (**1**) such as high efficiency, low catalyst loading, working under solvent-free conditions, short reaction time, and reusability make it superior to the most of similar reported protocols.

**Table 4:** Comparison of the catalytic activity of Cu@APS-TDU-PMO (**1**) with other reported catalytic systems.

Entry	Catalyst	Catalyst loading (mg)	Temperature (°C)	Time (min)	References
1	Nano-NiO	4.5	70	360	^122^
2	Fe_3_O_4_@APTMS-DFX	30	120	60	^123^
3	Cu-MCM-41	30	140	720	^124^
4	Mesoporous ZnS	91	120	36 h	^125^
5	Cu@APS-TDU-PMO (1)	30	110	30	This work

### Reusability of the Cu@APS-TDU-PMO nanocatalyst (1) for the synthesis of 2-(1*H*-tetrazol-5-yl) acrylonitrile derivative **5**

**Figure 9:**
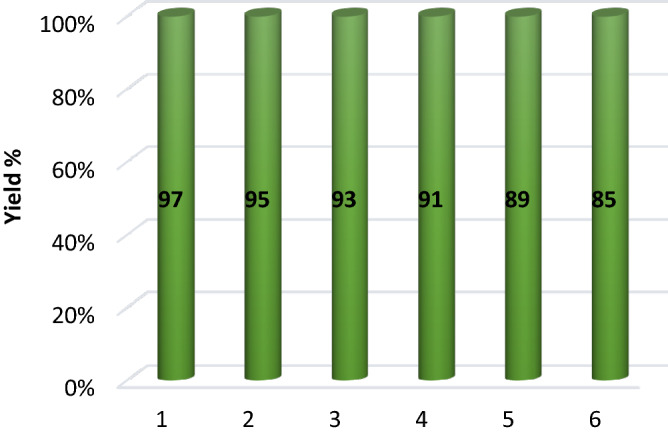
Reusability of the heterogeneous Cu@APS-TDU-PMO catalyst (**1**) for the synthesis of **5a**.

**Figure 10:**
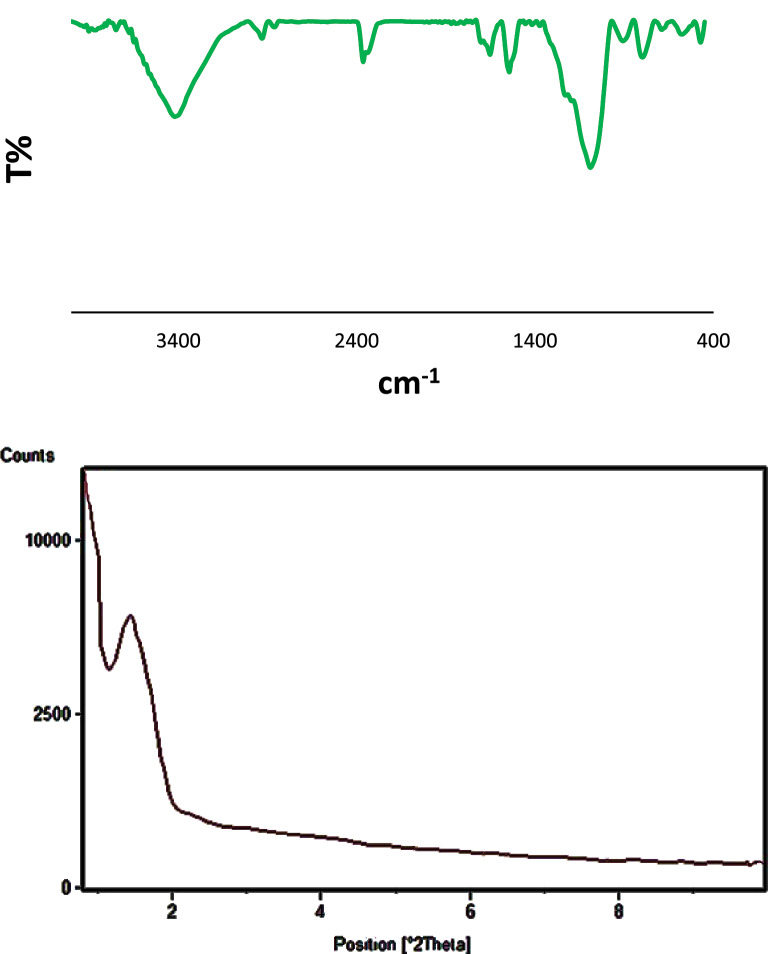
FTIR spectra and low angle XRD pattern of the reused Cu@APS-TDU-PMO catalyst (**1**) after six consecutive runs.

Performing chemical reactions using recyclable and reusable catalytic systems is a significant issue in terms of green chemistry and environmental protection principles. In this study, recyclability of the Cu@APS-TDU-PMO catalyst (**1**) was also investigated. For this purpose, the catalyst was separated from the reaction mixture using filtration, then washed with EtOH, and dried at 60 °C for 2 h. The recycled catalyst **1** in each step was used in six consecutive model reaction under optimal conditions for the synthesis of (*E*)-3-phenyl-2-(1*H*-tetrazol-5-yl)acrylonitrile derivative **5a**. As shown in Fig. 9, the catalytic activity of Cu@APS-TDU-PMO (1) was slightly decreased from 97 to 85%. To demonstrate the stability of the Cu@APS-TDU-PMO (1) under optimal conditions, the FTIR spectra and low angle XRD pattern of recycled catalyst after six consecutive runs have been presented in Fig. 10.

## Experimental section

### Materials and instrumentation

All chemicals were purchased from Merck or Aldrich and used as received, except for benzaldehyde which was distilled before its using. Characterization of the new Cu@APS-TDU-PMO (**1**) was performed by FESEM (TESCAN-MIRA3), TEM (Philips EM 208S), FTIR (Shimadzu 8400S), BET (ASAP™ 2020 Micromeritics), and TGA Bahr Company STA 504). XRD patterns of the mesoporous silica nanosphere were obtained using TW 1800 diffractometer with CuKα radiation (λ = 1.54050 Å). ^1^H NMR and spectra (500 MHz, Bruker DRX-500 Avance spectrometer) were recorded in DMSO-*d*_6_ at ambient temperature. Spectral data were compared with those obtained from authentic samples or reported in the literature. Distilled water was used in all experiments.

### General procedure for the preparation of 1,3-bis(3-(triethoxysilyl)propyl) urea bridge (**6**, APS-TDU)

**Figure 11:**
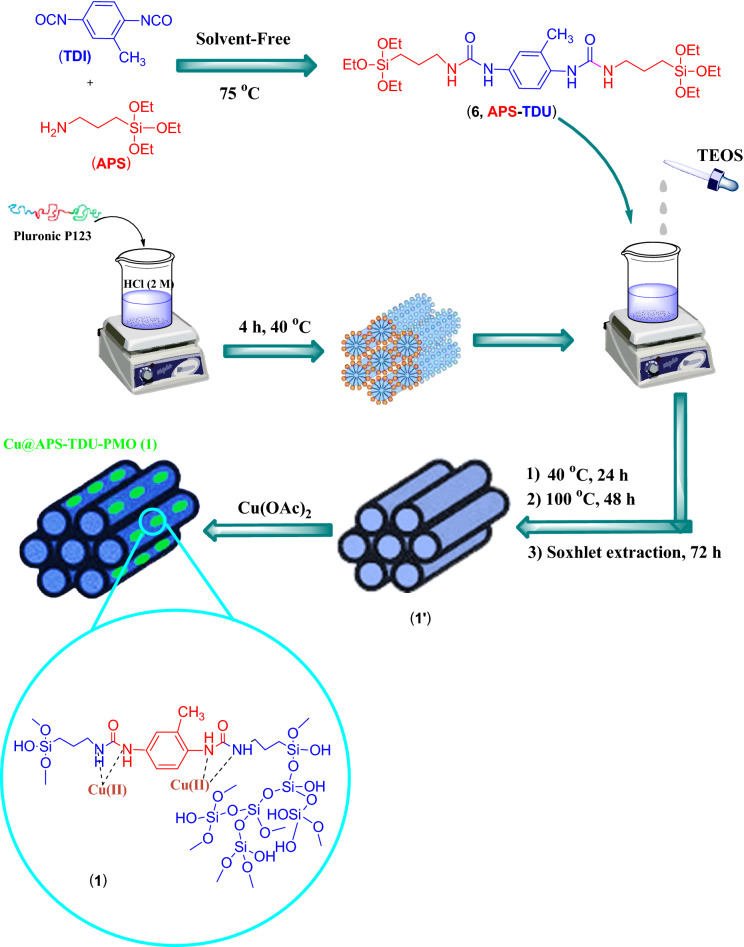
General procedure for the preparation of Cu@APS-TDU-PMO nanocatalyst (**1**).

First, (3-aminopropyl)triethoxysilane (APS, 6.16 g, 28.0 mmmol) was added dropwise to toluene-2,4-diisocyanate (TDI, 2.46 g, 14.0 mmmol) in a 50 mL round-bottom flask and the mixture was stirred under solvent-free condition at 75 °C for 4 h. Then, the mixture was cooled down to room temperature and stirred for 12 h to obtain a white gel. Subsequently, CHCl_3_ (10 mL) was added to the white gel and a clear solution was obtained. Then, hexane (10 mL) was added to the obtained solution and a white solid was precipitated, which was separated by filtration and washed with hexane and dried at 70 °C to give 6.5 g of 1,3-bis(3-(triethoxysilyl)propyl) urea bridge (**6**, Fig. 11).

### General procedure for the preparation of APS-TDU-PMO (**1′**)

P123 (4.0 g), as a surfactant, was dissolved in HCl (2.0 M, 150 mL) in a 250 mL round-bottom flask and the mixture was heated to 40 °C under stirring for 4 h. Then, 1,3-bis(3-(triethoxysilyl)propyl) urea bridge (**6**, 3.5 g) was dissolved in a solution of tetraethyl orthosilicate (TEOS, 11.09 g, 53.2 mmol) in CHCl_3_ (25 mL). The obtained solution was added dropwise to the solution of P123 and HCl and stirred for 24 h at 40 °C and then aging for 48 h at 100 °C. Eventually, the obtained white solid was washed with EtOH (10 mL) and hexane (10 mL) and dried at 80 °C. The surfactant was extracted under Soxhlet extraction conditions using EtOH-aqueous HCl for 72 h. Finally, the white solid was dried at 100 °C for 12 h to give 6.0 g of APS-TDU-PMO (**1′**, Fig. 11).

### General procedure for the preparation of Cu@APS-TDU-PMO (**1**)

Cu(OAc)_2_ (0.5 g, 2.8 mmol) was dissolved in 5.0 mL distilled water and the obtained solution was added slowly to the suspension of APS-TDU-PMO (0.5 g) in distilled water (10 mL). The obtained mixture was stirred at room temperature for 24 h. Finally, the resulting green solid was collected, washed with distilled water and EtOH, and then dried at 60 °C for 5 h to afford Cu@APS-TDU-PMO (**1**, 0.7 g, Fig. 11).

### General procedure for the preparation of 2-(1*H*-tetrazol-5-yl) acrylonitrile derivatives **5a–l**

Cu@APS-TDU-PMO (**1**, 30 mg), aromatic aldehyde (**2a–l**, 1.0 mmol), malononitrile (**3**, 1.0 mmol), and NaN_3_ (**4**, 1.20 mmol) were mixed, in a 10 mL round-bottom flask equipped with a magnetic stirrer and condenser, and then heated under solvent-free conditions to 110 °C. The reaction progress was monitored by TLC. After completion of the reaction, the reaction mixture was dispersed in HCl (2.0 M, 2 mL) and EtOAc (10 mL) was added and stirred for 15 min. Then, the solid catalyst **1** was separated by filtration and filtrate was extracted using EtOAc (5 mL). Finally, the solvent of collected organic layers was evaporated under reduced pressure on a rotary evaporator and the obtained solids were recrystallized in EtOH/H_2_O to afford the pure products **5a–l**. The recovered catalyst was reused after drying at 100 °C for 2 h for subsequent cycles.

### The FTIR, ^1^H NMR and ^13^C NMR data of selected tetrazole derivatives

#### (*E*)-3-(2-Chlorophenyl)-2-(1*H*-tetrazole-5-yl)acrylonitrile (**5b**)

FTIR (KBr disc): ῡ (cm^−1^), 3420 (NH), 2221 (C≡N), 1564 (C=C); ^1^H NMR (500 MHz, DMSO-*d*_6_): δ (ppm), 7.58–7.59 (2H, d, CH-Ar), 7.61–7.69 (1H, t, *J* = 7.2 Hz, CH-Ar), 8.13–8.14 (1H, d, CH-Ar), 8.54 (1H, s, CH), 13.22 (br s, NH); ^13^C NMR (125 MHz, DMSO-*d*_6_): δ (ppm), 80.14, 116.80, 129.17, 129.88, 130.73, 131.97, 134.39, 135.19, 147.04, 159.07, 161.37.

#### (*E*)-3‑(4‑Methoxyphenyl)‑2‑(1*H*‑tetrazole‑5‑yl)acrylonitrile (**5g**)

FTIR (KBr, disc): ῡ (cm^−1^), 3146 (NH), 2224 (C≡N), 1586 (C=C); ^1^H NMR (500 MHz, DMSO-*d*_6_): δ (ppm), 3.82 (3H, s, OCH_3_), 7.09–7.11 (1H, d, CH-Ar), 7.96–7.99 (1H, d, CH-Ar), 8.21 (1H, s, CH), 13.70 (br s, NH); ^13^C NMR (125 MHz, DMSO-*d*_6_): δ (ppm), 56.02, 93.70, 115.25, 116.55, 125.21, 132.61, 147.97, 155.85, 162.91.

## Conclusions

In conclusion, the supramolecular toluene-2,4-diurea-based periodic mesoporous organosilica containing Cu nanoparticles on its pore wall (Cu@APS-TDU-PMO) was synthesized for the first time. The prepared Cu@APS-TDU-PMO (**1**) was characterized by using FTIR, EDX, TGA, XRD, FESEM, BET, and TEM spectroscopic, microscopic or analytical methods and techniques. The Cu@APS-TDU-PMO showed unique characteristics such as porous structure with adjustable and uniform pore size distribution, high thermal stability and surface area. The new Cu@APS-TDU-PMO nanomaterial was efficiently used as a promising and recyclable catalyst for the synthesis of different 2-(1*H*-tetrazol-5-yl)acrylonitrile derivatives through multicomponent strategy under solvent-free conditions. In addition, the catalyst can be easily separated by filtration and reused several times without significant loss of its catalytic activity.

## Supplementary Information

Supplementary Information.

## Data Availability

All data generated or analyzed during this study are included in this published article [and its supplementary information files].
