# Supplementary material for: Design and characterization of an urea-bridged PMO supporting Cu(II) nanoparticles as highly efficient heterogeneous catalyst for synthesis of tetrazole derivatives
Source: Sci Rep. 2022 Oct 28;12:18139. doi: 10.1038/s41598-022-22905-7 (PMC9616949; doi:10.1038/s41598-022-22905-7)
Supplement: Supplementary file 1 — Supplementary Information. [file 41598_2022_22905_MOESM1_ESM.docx]

**Electronic Supporting Information**

**Design and characterization of an urea-bridged PMO supporting Cu(II) nanoparticles as highly efficient heterogeneous catalyst for synthesis of tetrazole derivatives**

*Ehsan Valiey and Mohammad G. Dekamin**

*^a^Pharmaceutical and Heterocyclic Compounds Research Laboratory, Department of Chemistry, Iran University of Science and Technology, Tehran, 16846-13114, Iran.*

**E-mail:* [*mdekamin@iust.ac.ir*](mailto:mdekamin@iust.ac.ir)

| **Page** | **Contents** |
| --- | --- |
| S1 | Title page |
| S2 | General procedure for the preparation of 1,3-bis(3-(triethoxysilyl)propyl) urea bridge (APS-TDU, **6**) |
| S2 | General procedure for the preparation of APS-TDU-PMO (**1′**) |
| S2 | General procedure for the preparation of Cu@APS-TDU-PMO (**1**) |
| S4 | Characterization of the Cu@APS-TDU-PMO (**1**) |
| S9 | General procedure for the preparation of 2-(1*H*-tetrazol-5-yl) acrylonitrile derivatives **5a-l** |
| S10 | Characterization of (*E*)-3-(2-chlorophenyl)-2-(1*H*-tetrazole-5-yl)acrylonitrile (**5b**) |
| S13 | Characterization of (*E*)-3‑(4‑methoxyphenyl)‑2‑(1*H*‑tetrazole‑5‑yl)acrylonitrile (**5g**) |

**General procedure for the preparation of 1,3-bis(3-(triethoxysilyl)propyl) urea bridge (6**, **APS-TDU)**

First, (3-aminopropyl)triethoxysilane (APS, 6.16 g, 28.0 mmmol) was added dropwise to toluene-2,4-diisocyanate (TDI, 2.46 g, 14.0 mmmol) in a 50 mL round-bottom flask and the mixture was stirred under solvent-free condition at 75 °C for 4 h. Then, the mixture was cooled down to room temperature and stirred for 12 h to obtain a white gel. Subsequently, CHCl_3_ (10 mL) was added to the white gel and a clear solution was obtained. Then, hexane (10 mL) was added to the obtained solution and a white solid was precipitated, which was separated by filtration and washed with hexane and dried at 70 °C to give 6.5 g of 1,3-bis(3-(triethoxysilyl)propyl) urea bridge (**6**, **Figure S1**).

**General procedure for the preparation of APS-TDU-PMO (1′)**

P123 (4.0 g), as a surfactant, was dissolved in HCl (2.0 M, 150 mL) in a 250 mL round-bottom flask and the mixture was heated to 40 °C under stirring for 4 h. Then, 1,3-bis(3-(triethoxysilyl)propyl) urea bridge (**6**, 3.5 g) was dissolved in a solution of tetraethyl orthosilicate (TEOS, 11.09 g, 53.2 mmol) in CHCl_3_ (25 mL). The obtained solution was added dropwise to the solution of P123 and HCl and stirred for 24 h at 40 °C and then aging for 48 h at 100 °C. Eventually, the obtained white solid was washed with EtOH (10 mL) and hexane (10 mL) and dried at 80 °C. The surfactant was extracted under Soxhlet extraction conditions using EtOH-aqueous HCl for 72 h. Finally, the white solid was dried at 100 °C for 12 h to give 6.0 g of APS-TDU-PMO (**1′**, **Figure S1**).

**General procedure for the preparation of Cu@APS-TDU-PMO (1)**

Cu(OAc)_2_ (0.5 g, 2.8 mmol) was dissolved in 5.0 mL distilled water and the obtained solution was added slowly to the suspension of APS-TDU-PMO (0.5 g) in distilled water (10 mL). The obtained mixture was stirred at room temperature for 24 h. Finally, the resulting green solid was collected, washed with distilled water and EtOH, and then dried at 60 °C for 5 h to afford Cu@APS-TDU-PMO (**1**, 0.7 g, **Figure S1**).

**Figure S1.** Schematic procedure for the preparation of Cu@APS-TDU-PMO nanocatalyst (**1**).

**Characterization of the Cu@APS-TDU-PMO (1):**


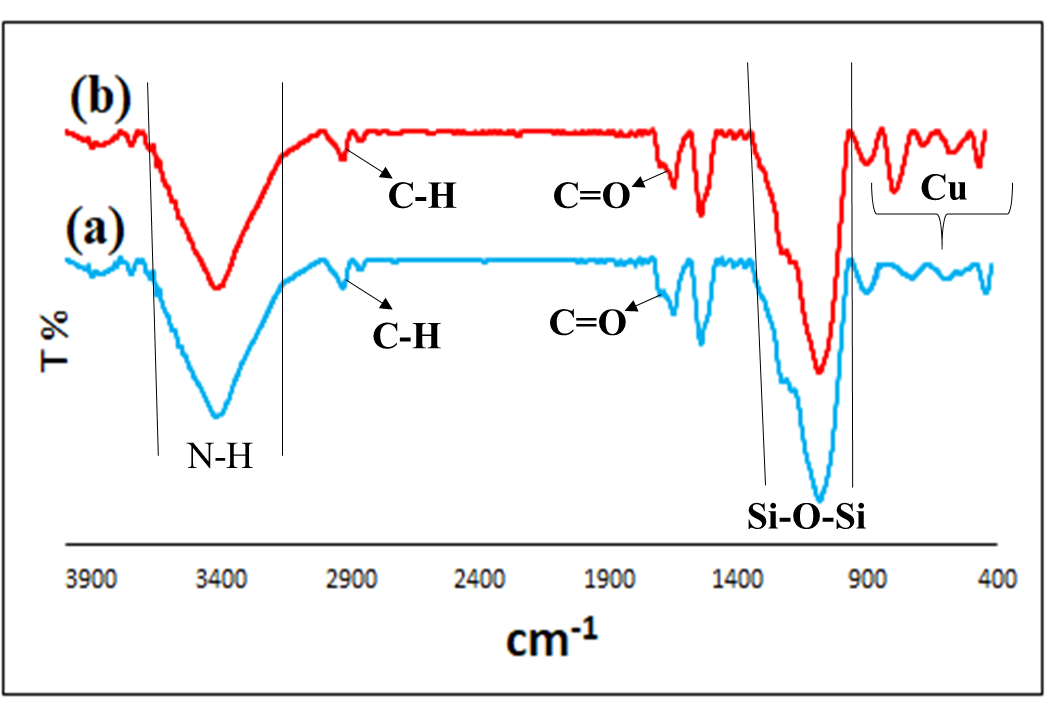


**Figure S2.** FTIR spectra of the APS-TDU-PMO (**a**) and Cu@APS-TDU-PMO (**1**, **b**).


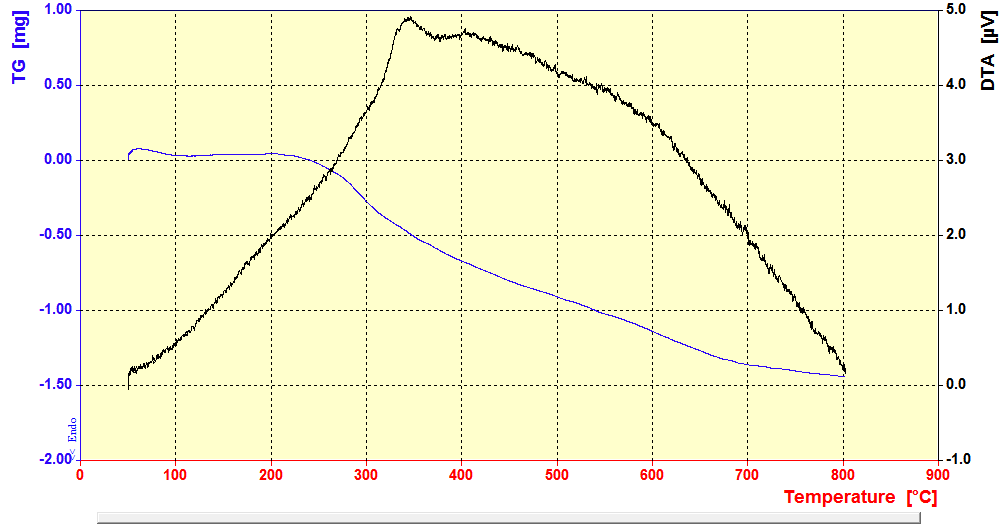


**Figure S3.** TGA curve of the Cu@APS-TDU-PMO nanomaterial (**1**).

| 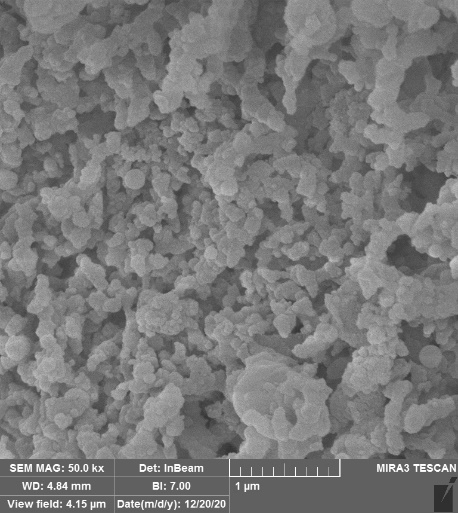  **(a)** | 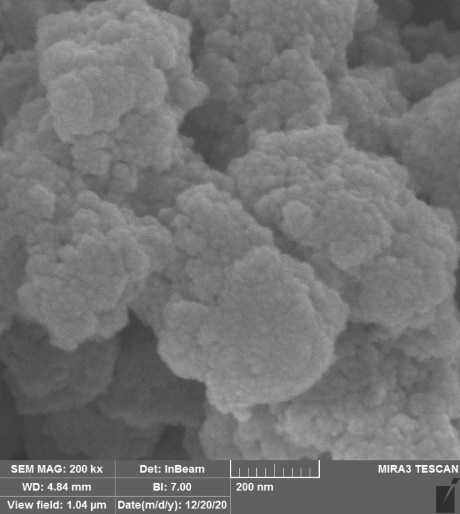  **(b)** | 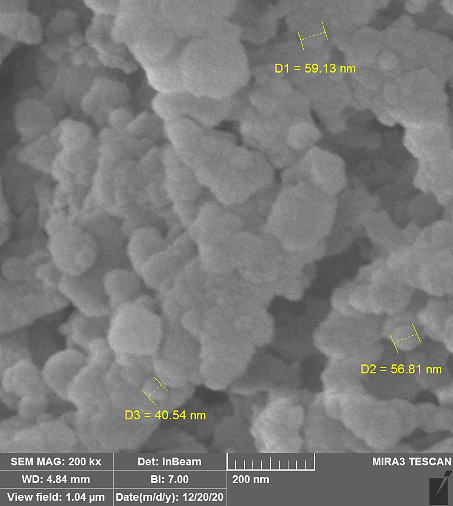  **(c)** |
| --- | --- | --- |
| 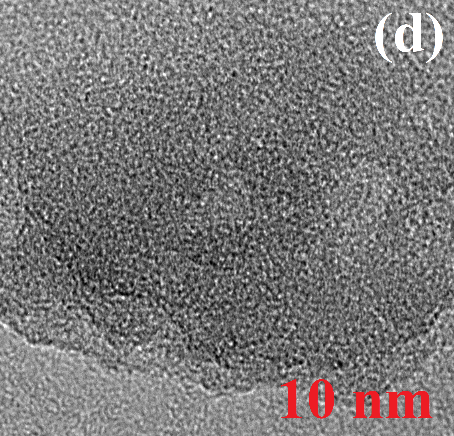 | 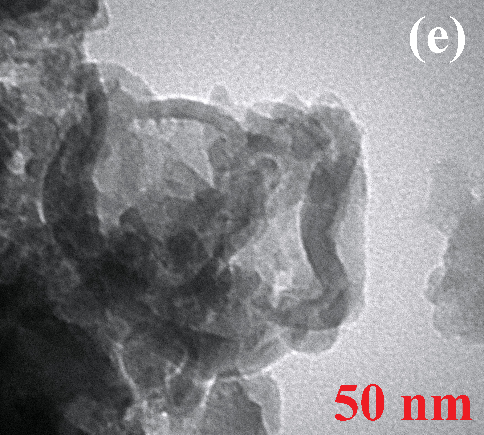 | 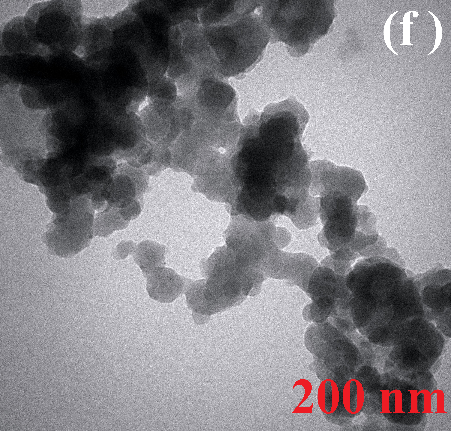 |

**Figure S4.**  FESEM (**a**, **b**, **c**) and TEM images (**d**, **e**, **f**) of the Cu@APS-TDU-PMO nanoreactor (**1**).


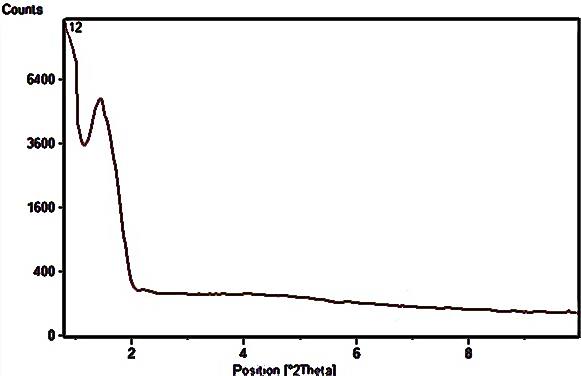


**(a)**

**(b)**


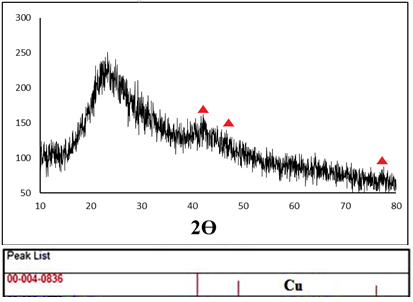


**(c)**

**Figure S5.** Low-angle (**a**) and wide angle (**b**) XRD patterns of the APS-TDU-PMO; Wide angle XRD pattern of the Cu@APS-TDU-PMO (**1, c**).


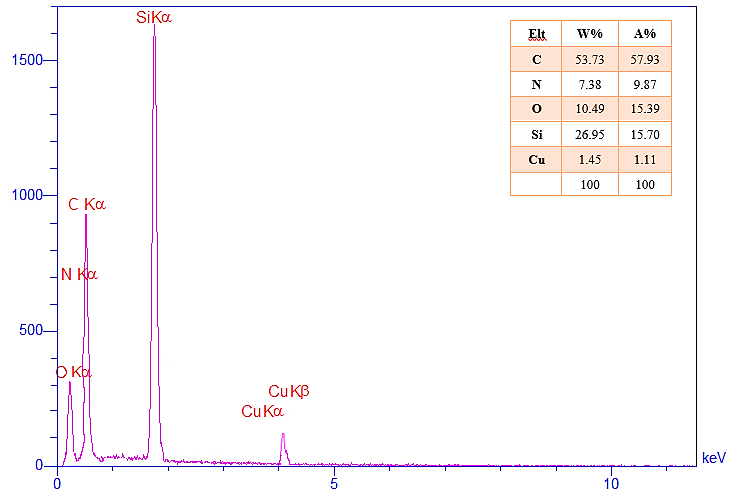


**Figure S6.** EDX analysis of the Cu@APS-TDU-PMO nanocomposite (**1**).

**Table 1** Structural parameters of the Cu@APS-TDU-PMO (**1**) determined from N_2_ adsorption-desorption experiments.

| Sample | Pore diameter (nm) | Surface area (m^2^ g^-1^) | Vp (cm^3^ g^-1^) |
| --- | --- | --- | --- |
| Cu@APS-TDU-PMO (1) | 5.74 | 276 | 0.17 |


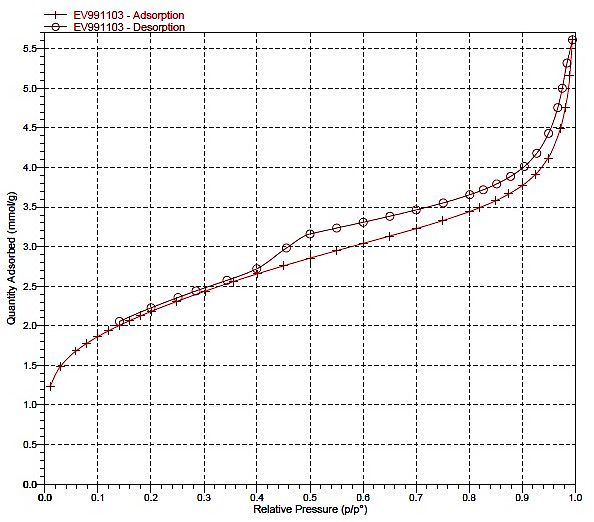


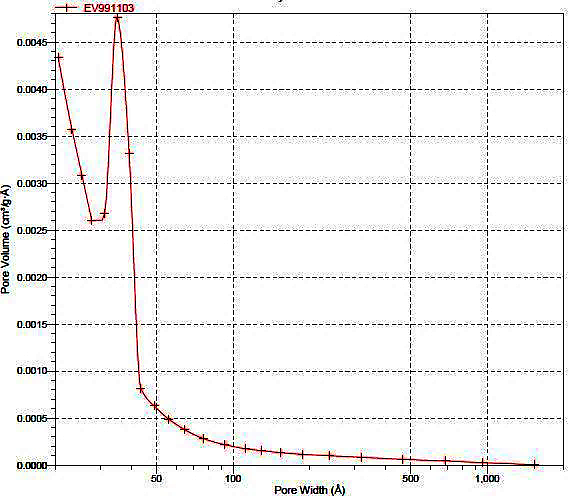


**Figure S7.** . N_2_ adsorption-desorption isotherm of the Cu@APS-TDU-PMO mesoporous material (**1**).

**General procedure for the preparation of 2-(1*H*-tetrazol-5-yl) acrylonitrile derivatives 5a-l**

Cu@APS-TDU-PMO (**1**, 30 mg), aromatic aldehyde (**2a-l**, 1.0 mmol), malononitrile (**3**, 1.0 mmol), and NaN_3_ (**4**, 1.20 mmol) were mixed, in a 10 mL round-bottom flask equipped with a magnetic stirrer and condenser, and then heated under solvent-free conditions to 110 °C. The reaction progress was monitored by TLC. After completion of the reaction, the reaction mixture was dispersed in HCl (2.0 M, 2 mL) and EtOAc (10 mL) was added and stirred for 15 min. Then, the solid catalyst **1** was separated by filtration and filtrate was extracted using EtOAc (5 mL). Finally, the solvent of collected organic layers was evaporated under reduced pressure on a rotary evaporator and the obtained solids were recrystallized in EtOH/H_2_O to afford the pure products **5a-l**. The recovered catalyst was reused after drying at 100 °C for 2 h for subsequent cycles.

**The FTIR, ^1^H NMR and ^13^C NMR data of selected tetrazole derivatives**

(*E*)-3-(2-Chlorophenyl)-2-(1*H*-tetrazole-5-yl)acrylonitrile (**5b**):

FTIR (KBr disc): ῡ (cm^-1^), 3420 (NH), 2221 (C≡N), 1564 (C=C); ^1^H NMR (500 MHz, DMSO-*d*_6_): δ (ppm), 7.58 –7.59 (2H, d, CH-Ar), 7.61 – 7.69 (1H, t, *J* = 7.2 Hz, CH-Ar), 8.13 – 8.14 (1H, d, CH-Ar), 8.54 (1H, s, CH), 13.22 (br s, NH); ^13^C NMR (125 MHz, DMSO-*d*_6_): δ (ppm), 80.14, 116.80, 129.17, 129.88, 130.73, 131.97, 134.39, 135.19, 147.04, 159.07, 161.37.

**Figure S8.** FTIR spectra of (*E*)-3-(2-chlorophenyl)-2-(1*H*-tetrazole-5-yl)acrylonitrile (**5b**).

**
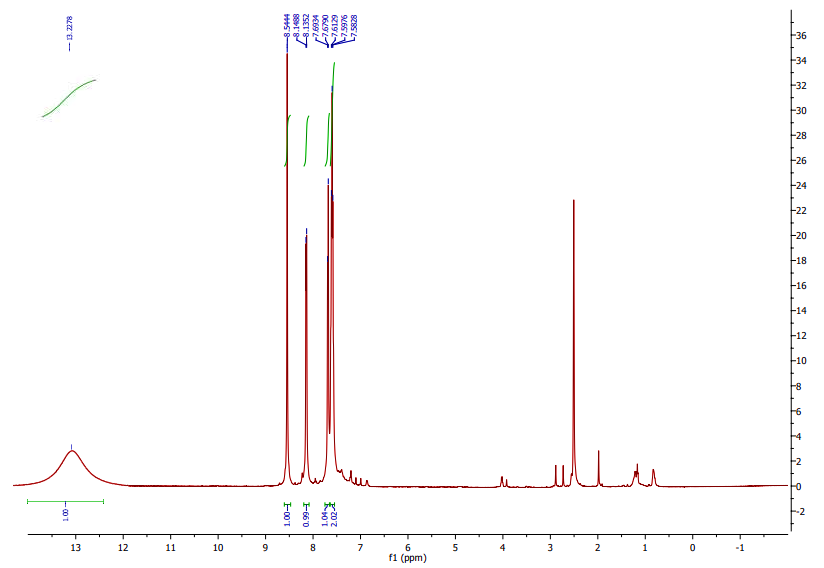
**

**Figure S9.** ^1^H NMR spectra of (*E*)-3-(2-chlorophenyl)-2-(1*H*-tetrazole-5-yl)acrylonitrile (**5b**).

**
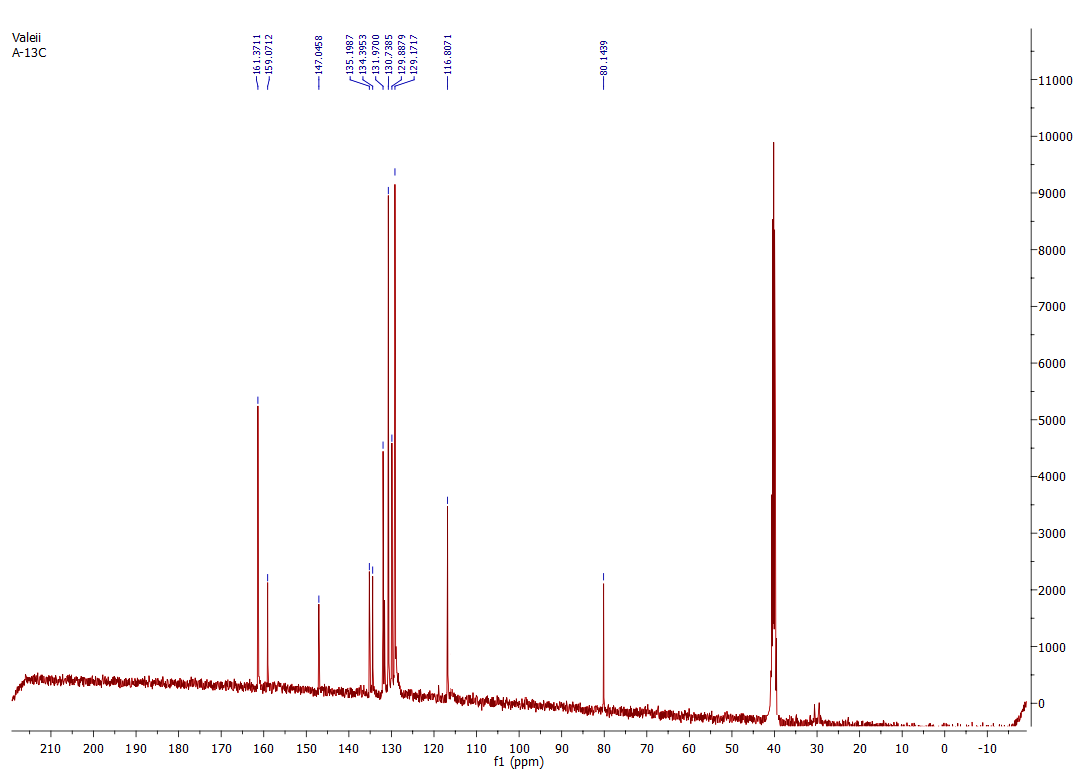
**

**Figure S10.** ^13^C NMR spectra of (*E*)-3-(2-chlorophenyl)-2-(1*H*-tetrazole-5-yl)acrylonitrile (**5b**).

(*E*)-3‑(4‑Methoxyphenyl)‑2‑(1*H*‑tetrazole‑5‑yl)acrylonitrile (**5g**):

FTIR (KBr, disc): ῡ (cm^-1^), 31**46** (NH), 222**4** (C≡N), 15**86** (C=C); ^1^H NMR (500 MHz, DMSO-*d*_6_): δ (ppm), 3.82 (3H, s, OCH_3_), 7.09 – 7.11 (1H, d, CH-Ar), 7.96 – 7.99 (1H, d, CH-Ar), 8.21 (1H, s, CH), 13.70 (br s, NH); ^13^C NMR (125 MHz, DMSO-*d*_6_): δ (ppm), 56.02, 93.70, 115.25, 116.55, 125.21, 132.61, 147.97, 155.85, 162.91.

**Figure S11.** FTIR spectra of (*E*)-3‑(4‑methoxyphenyl)‑2‑(1*H*‑tetrazole‑5‑yl)acrylonitrile (**5g**).


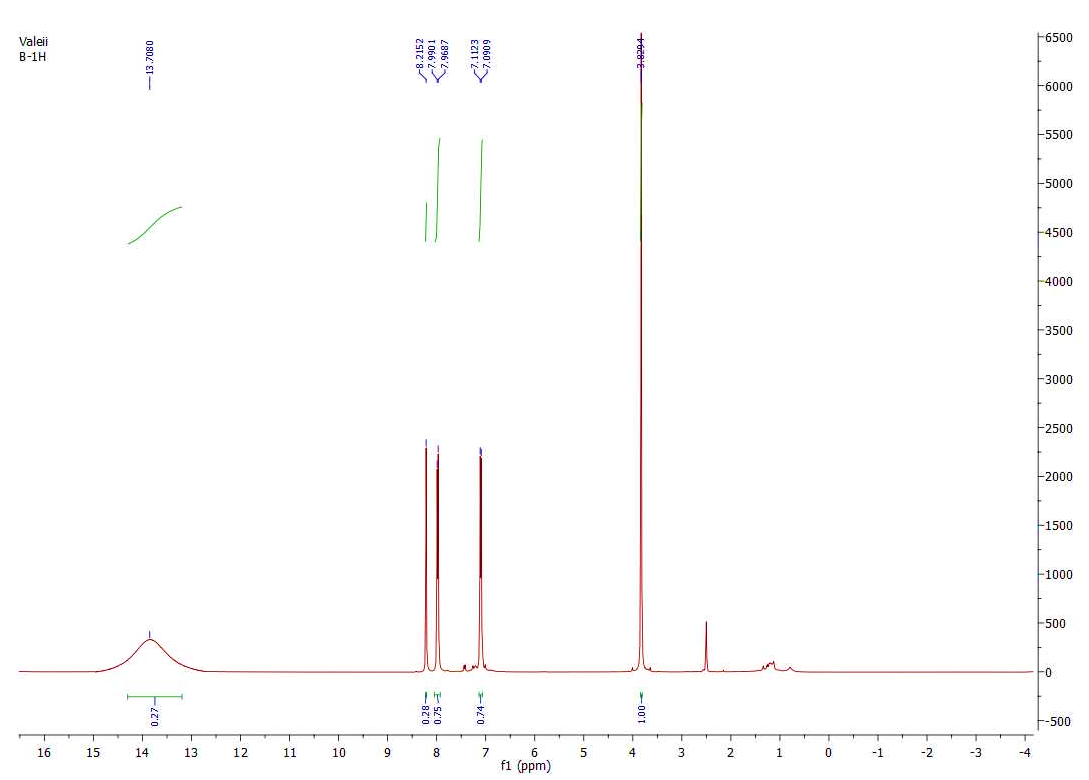


**Figure S12.** ^1^H NMR spectra of (*E*)-3‑(4‑methoxyphenyl)‑2‑(1*H*‑tetrazole‑5‑yl)acrylonitrile (**5g**).


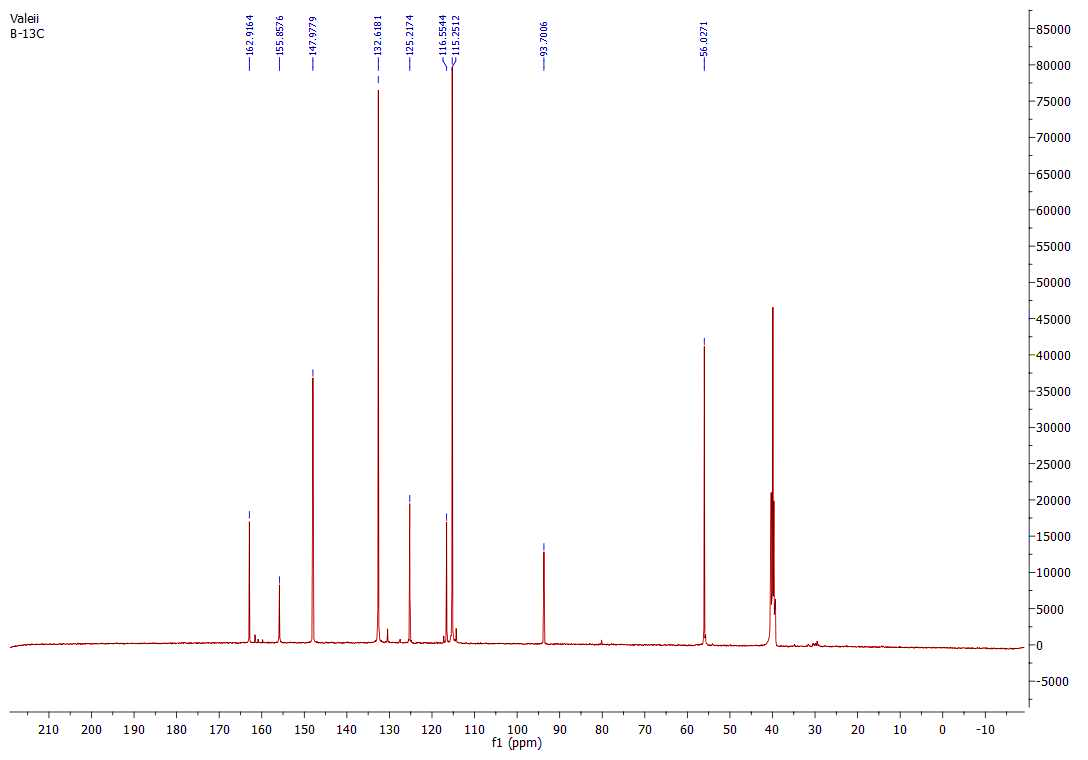


**Figure S13.** ^13^C NMR spectra of (*E*)-3‑(4‑methoxyphenyl)‑2‑(1*H*‑tetrazole‑5‑yl)acrylonitrile (**5g**).
